# Study of Post-Diphtheritic Paralyses

**Published:** 1892-12

**Authors:** 


STUDY OF POST-DIPHTHERITIC PARALYSES.—Baginsky (*International Medical Magazine*) believes that the more intense the diphtheritic process in the pharynx, the earlier does paralysis follow. Paralysis of the soft palate is the most usual, and appears consecutively with albuminuria or well-delivered nephritis. The heart is often affected early, and the children die with symptoms of heart weakness.

Paralysis appearing later and with slower onset, is associated with non-gangrenous or non-septic processes. Generalization of the paralysis, or especially its localization to the diaphragm, is dangerous. Paralysis of the diaphragm is of more frequent occurrence than is generally believed. This is characterized by almost complete aphonia, cough, difficult and copious expectoration, foamy, viscid mucous, dyspnœa with thoracic respiration. The affection is usually fatal, death occurring slowly with asphyxia, bronchitis, or broncho-pneumonia, or suddenly with complete cessation of respiration.

The heart manifestations are manifold, varying from diminution in arterial tension to arlythenia, with symptoms of stasis. On auscultation, the first sound is absent, both sounds indistinct or first sound reduplicated. Accompanying this may be a rapid and extensive swelling of the liver. This is of grave prognosis. Cheyne-Stokes respiration may occur. Recovery may take place after all these symptoms have appeared.

The best results in treatment, obtained by Baginsky, have been from the subcutaneous injection of sulphate of strychnia, 1-20 to 1-40 of a grain a day in three injections. Camphor hypodermically has also given good results.

He accepts as the pathological basis for diphtheritic paralysis, a polyneuritis, or as Virchow has expressed it, "*neuritis parenchymatosa et interstitialis proliferans.—Annals of Gynecology and Pœdiatry.*

